# Mutations in the TERC template sequence can be incorporated into the telomeres of human tumors

**DOI:** 10.1371/journal.pone.0272707

**Published:** 2022-08-25

**Authors:** Radwa Sharaf, Garrett M. Frampton, Lee A. Albacker

**Affiliations:** Foundation Medicine Inc., Cambridge, MA, United States of America; University of Nebraska Medical Center, UNITED STATES

## Abstract

Telomerase-mediated lengthening is a mechanism by which some cancer cells avoid senescence-mediated cell cycle arrest due to shortened telomeres. By reverse transcribing an RNA template, encoded by *TERC*, the enzyme telomerase synthesizes the elongation of telomeric DNA using the 3’ end of the chromosome as a primer. *TERC* harbors a highly conserved template region consisting of 11 nucleotides spanning hg19 coordinates chr3:169482793–169482803. In human cell lines, when *TERC* was mutated to alter its template region, telomerase generated the predicted mutant telomeric repeats. However, it is unknown if this can occur in human clinical samples. Here, we report on the rare occurrence of two tumor samples where *TERC* template mutations were reflected in telomeric repeats.

## Introduction

Telomeres are protective DNA-protein complexes present at the ends of linear chromosomes, consisting of repetitive hexamers (TTAGGG) that shorten in length with every cell division by an average of 50–150 base pairs [1, 2]. If telomere length falls below a critical threshold, cells undergo senescence. In order to allow for infinite proliferation, tumor cells must overcome the telomere shortening problem [3]. Telomerase-mediated lengthening is a telomere maintenance mechanism, which relies on the overexpression of the telomerase enzyme, encoded by *TERT*, and was observed in 85–90% of tumors [4, 5]. By reverse transcribing *TERC*, which encodes an RNA template, the telomerase enzyme can synthesize the elongation of telomeric DNA using the 3’ end of the chromosome as a primer [6, 7].

*TERC* is a lncRNA harboring a highly conserved template region consisting of 11 nucleotides spanning hg19 coordinates chr3:169482793–169482803 encoding GTTAGGGTTAG [8, 9]. Positions 1–5 basepair with the end of the telomere and positions 6–11 are reverse transcribed into new telomeric sequence [10, 11]. In human cell lines, when *TERC* was mutated to alter its template region, telomerase generated the predicted mutant telomeric repeats [12–14]. However, this has never been demonstrated previously in human clinical samples. Here, we report two tumor samples where *TERC* template mutations were reflected in telomeric repeats.

## Results and discussion

**Fig 1:**
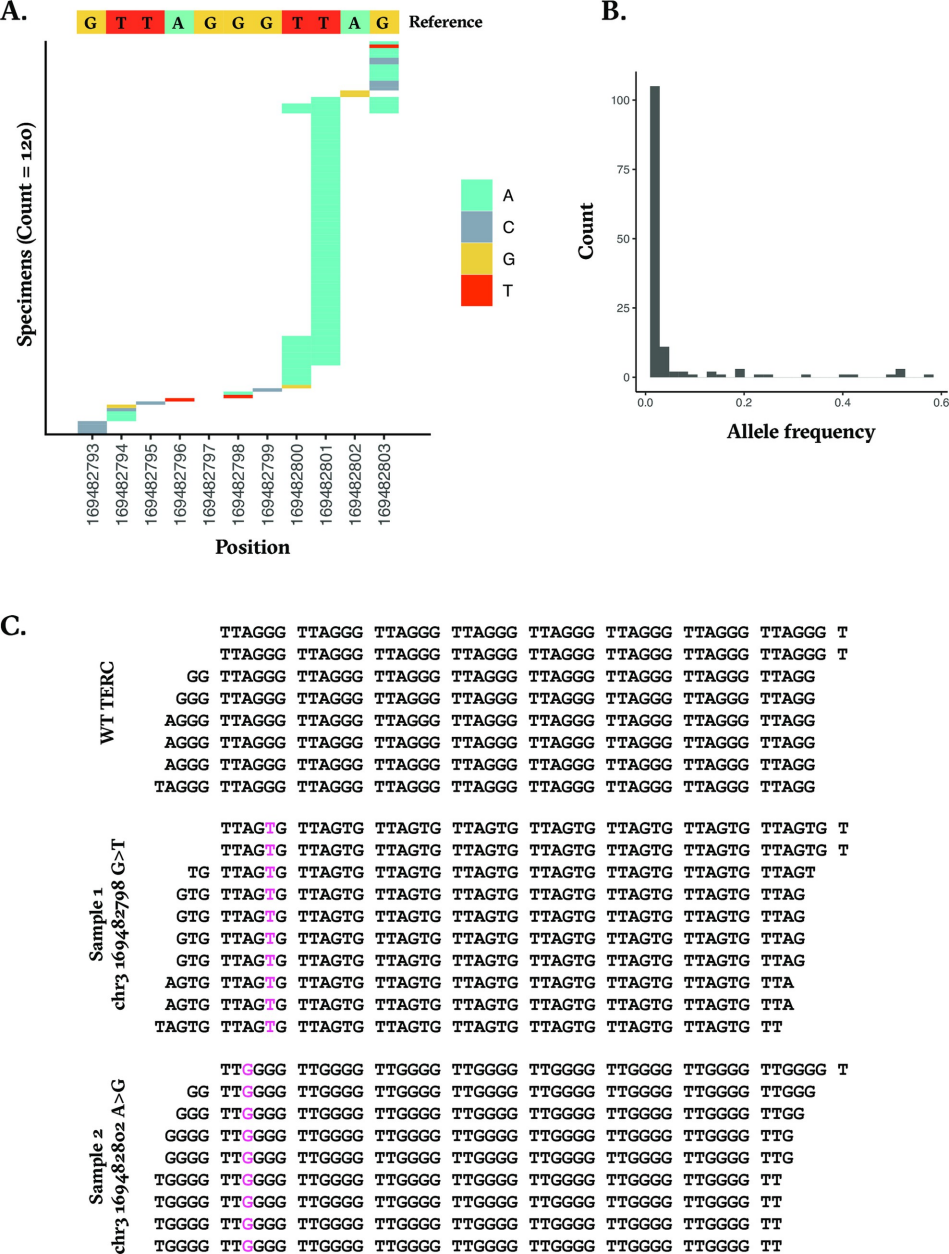
**A.** Figure depicting the positions along TERC’s template sequence that were mutated in samples across Foundation Medicine’s dataset. **B.** A histogram of the allele frequencies of the *TERC* template mutations detected. **C.** Telomeric reads detected in samples harboring WT *TERC*, a 169482798 G>T *TERC* mutation, or a 169482802 A>G *TERC* mutation.

Across 309,384 unique tumor samples from the Foundation Medicine dataset, we identified 120 samples carrying 137 mutations in *TERC*’s template sequence (**Fig 1A**). We observed an over-representation of mutations at chr3:169482801 (position 9), seen in 68% (82) of samples with a mutation. Overall, 88% of samples harbored only one mutation in *TERC*’s template region, but in 12% of samples, multiple mutations in the template sequence were observed, where one mutation always impacted chr3:169482801 (position 9) and the other mutation was either at chr3:169482800 (position 8), or chr3:169482803 (position 11), or both. Interestingly, 86% (118) of the mutations identified were a transition from the reference base to an A. Of note, the observed allele frequency of these *TERC* template mutations was low, with a median of 0.02 (**Fig 1B**), indicating that only a small proportion of tumor cells across these samples carry a *TERC* template mutation.

**Fig 2:**
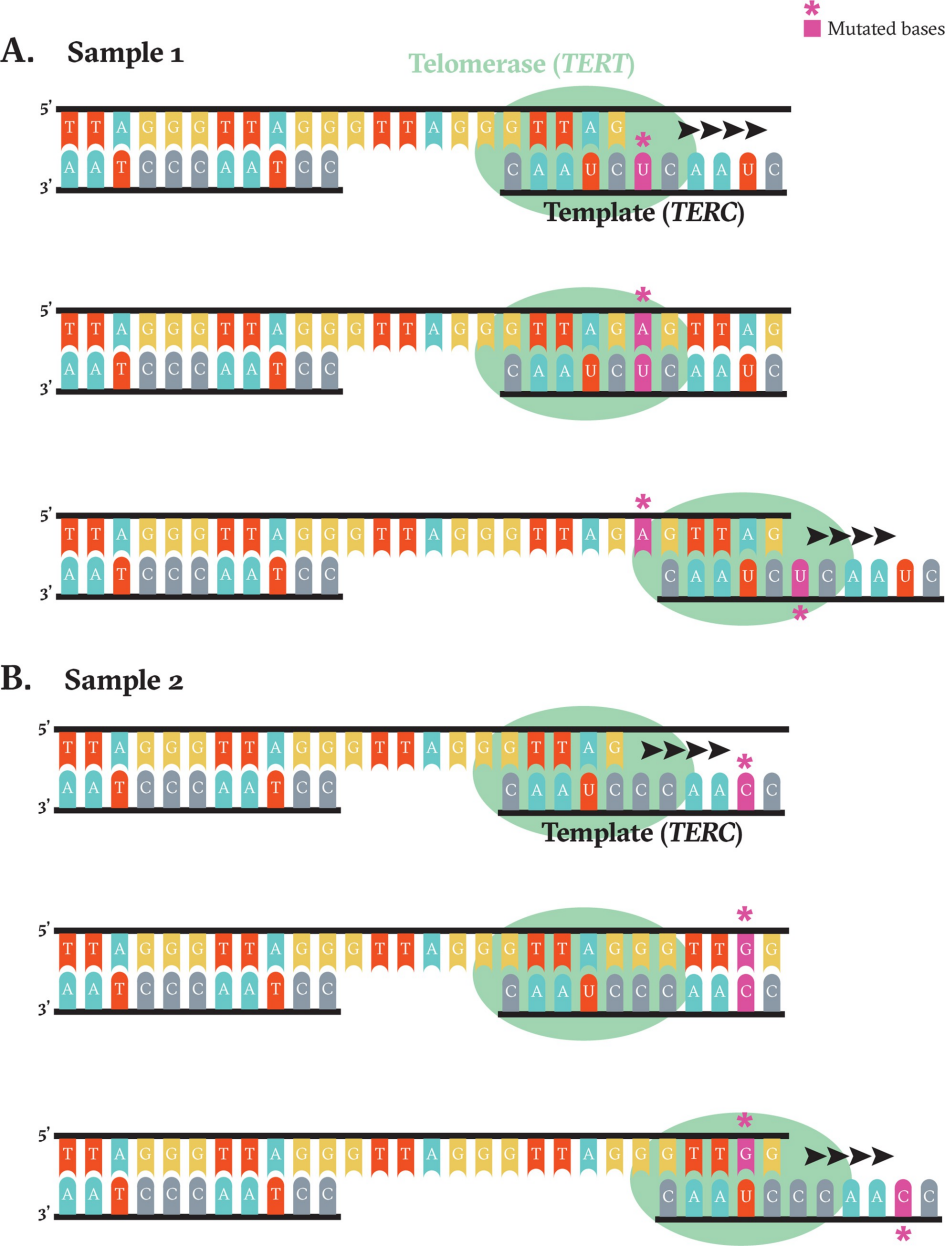
A model illustrating the impact of *TERC*‘s template mutations on the synthesis of telomeric repeats in sample 1 harboring a 169482798 G>T mutation (**A**) and sample 2 harboring a 169482802 A>G (**B**).

We then checked for the presence of mutated telomeric reads that matched the mutation of each sample’s *TERC* template sequence and found two samples that fit this criterion (**Fig 1C**). The allele frequency of the *TERC* mutation in both samples was in the top 90^th^ percentile (0.23 and 0.16). Sample 1 had a G > T mutation at chr3:169482798 (position 6) on the positive strand, which results in a C > U mutation in the middle of *TERC*’s template sequence. This mutation results in the priming of the synthesis of telomeric ends with TTAGTG repeats, instead of the canonical TTAGGG repeats (**Fig 1C**). Sample 1 is an ovary epithelial carcinoma and had an amplification in *RAD21*, which we have previously shown to be associated with increased telomeric content [15]. The telomeric content measured for sample 1 was 1330.3 telomeric reads per GC-content matched million reads (TRPM), which lies at the 65^th^ percentile across all 2623 ovary epithelial carcinoma samples in our cohort and at the 62^nd^ percentile of RAD21-altered ovary epithelial carcinomas. Sample 2 harbored an A > G mutation at chr3:169482802 (position 10) in *TERC*’s template sequence, which results in the synthesis of TTGGGG repeats instead of TTAGGG (**Fig 1C**). Sample 2 is a skin melanoma sample and harbored a *TERT* promoter mutation (-124 C > T), associated with upregulation of telomerase expression. The telomeric content of sample 2 was 1281.9 TRPM, at the 64^th^ percentile of all 5204 skin melanoma samples in our cohort and at the 67^th^ percentile of *TERTp*-mutated melanomas. As shown in Fig 2, the occurrence of altered telomeric sequence was observed across entire reads, where the mutated telomere variant repeats (TVRs) accounted for 0.63% of TVRs of sample 1 and 0.56% of TVRs in sample 2. From these data, we concluded that the altered template sequences were incorporated into telomeres in these samples.

Mechanistically, positions 1–5 of the *TERC* template sequence must basepair with the sequence transcribed from positions 7–11 to enable further elongation. Sample 1 had a mutation at position 6, which avoids this guide/template constraint (**Fig 2A**). Sample 2 had an alteration at position 10, which would necessitate a G:U pairing between the mutated telomeric repeat and the unmutated position 4 of *TERC*’s template sequence (**Fig 2B**). We hypothesize that in this case, wobble-base pairing allows for binding and priming of telomeric elongation [16, 17]. We also speculate that the presence of comutations associated with increased telomerase activity (*pTERT* and *RAD21*), played a role in incorporating mutated guide sequences into telomeric repeats.

In our dataset, the prevalence of *TERC* template mutations was quite low (0.04%). This is consistent with the observation that the expression of mutant template RNA in cancer cell lines negatively impacts cellular survival and proliferation [14, 18, 19]. Others have shown that mutated template sequence increases the sensitivity of cancer cell lines to a variety of chemotherapeutic drugs [20]. Similarly, in *Saccharomyces cerevisiae* and *Tetrahymena thermophila*, mutations of the template sequence resulted in mutated telomeres, but negatively impacted cell survival [21–23]. Our work demonstrates that mutations in the *TERC* template sequence exist in a small fraction of clinical tumor samples and rarely, this mutated template sequence can prime the synthesis of mutated telomeric repeats in clinical tumor samples.

## Methods

### Ethics approval and consent to participate

Approval for this study, including a waiver of informed consent and a HIPAA waiver of authorization, was obtained from the Western Institutional Review Board (Protocol No. 20152817). Consented data that can be released is included in the paper. Institutional review board approval of the study protocol was obtained prior to study conduct and included a waiver of informed consent.

### Sample sequencing

The Foundation Medicine dataset consisted of specimens sequenced as a part of routine clinical care using a targeted next-generation sequencing assay as previously described (FoundationOne CDx) [24, 25]. The pathologic diagnosis of each case was first made in the referring center and was then confirmed in our facility (Foundation Medicine, Cambridge MA). All samples contained a minimum of 20% tumor nuclei. The samples were assayed by adaptor ligation hybrid capture, performed for all coding exons of 309 cancer‐related genes plus select introns from 34 genes frequently rearranged in cancer. Sequencing of captured libraries was performed using the Illumina sequencing platform to a mean exon coverage depth for targeted regions of >500X, and sequences were analyzed for genomic alterations by an automated pipeline.

### Telomeric reads

For each sample, we generated a list of possible read sequences where the read exclusively consisted of TTAGGG repeats, except that the nucleotides mutated in *TERC*’s template were reflected in the repeat sequence.
